# Novel method to delineate palatal rugae and assess their complexity using fractal analysis

**DOI:** 10.1038/s41598-022-25910-y

**Published:** 2022-12-16

**Authors:** Miltiadis A. Makrygiannakis, Heleni Vastardis, Athanasios E. Athanasiou, Demetrios J. Halazonetis

**Affiliations:** 1 Department of Orthodontics, School of Dentistry, National and Kapodistrian University of Athens, 2 Thivon Str., 11527 Athens, Greece; 2 Department of Dentistry, European University Cyprus, Nicosia, Cyprus; 3 Hamdan Bin Mohammed College of Dental Medicine, Mohammed Bin Rashid University of Medicine and Health Sciences, Dubai, United Arab Emirates

**Keywords:** Diagnosis, Dentistry, Forensic dentistry, Orthodontics

## Abstract

Palatal rugae constitute significant morphological landmarks, with wide clinical applications in forensics, insertion of mini-screws, and superimposition. Their morphology has been studied mainly with indices relevant to their qualitative characteristics. The present paper aims at presenting a new quantitative method to evaluate their complexity, by means of box-counting fractal dimension analysis, and to investigate its inter- and intra-rater reliability. Twenty maxillary plaster models were scanned for the needs of this study. A sequence of steps, including cropping of the mesh, ball pivoting, distance mapping and fractal dimension analysis, performed with Viewbox 4 software, was followed. Box-counting fractal dimensions were calculated as a measure of rugae’s complexity. Inter- and intra-rater reliability were investigated, using Bland–Altman analysis. Fractal dimensions ranged from 1.274 to 1.491 (average: 1.412). Bland–Altman analysis of inter- and intra-examiner reliability demonstrated that the 95% limits of agreement ranged from − 0.012 to 0.011 and from − 0.004 to 0.004, respectively. The method is reliable and can be applied in research and forensics. It offers comprehensive evaluation of the rugae’s complexity and a complete set of information about their outlines and height profiles, with minimum user intervention.

## Introduction

The plicae palatinae, or palatal rugae, constitute transverse uneven ridges of mucosa, behind the incisive papilla, on the hard palate of most mammalian species^1,2^. They are located in the anterior part of the hard palate lateral to the midpalatal suture without ever crossing it^3^. Their number and arrangement are species-specific^1^. For example, pigs usually have 21 pairs of rugae, while humans and mice, up to 4 and 8, respectively^4^. Among the elements contained in the core of palatal rugae—namely glycosaminoglycans, elastic tissue and collagen—glycosaminoglycans play an integral role in maintaining rugae’s shape throughout life, due to their hydrophilic nature^5,6^.

Along with the teeth and tongue, rugae participate in mastication by contributing to sensing, holding and mashing the food^7^. In fact, rugae carry various types of intraepithelial sensory structures^8^ and participate in receiving sensory information when the food is pressed against the hard palate^9^. Furthermore, the mechanical function of facilitating mastication and preventing cutting down of a bolus is undertaken in animals, such as ruminants, whose rugae are prominent^10^.

Regarding relevant clinical applications, palatal rugae are significant morphological landmarks. They can be used as superimposition references because of their anatomical stability; especially that of the area around the 3rd ruga^11^. Furthermore, rugae can serve as anatomical guides for the insertion of orthodontic mini-screws in the palatal area^12^. Moreover, they can be used for post-mortem identification, when other forensic methods (fingerprints, DNA, dental records, etc.) are insufficient or fail. As they are protected by the teeth, lips and tongue, they can greatly endure the physical conditions accompanied by natural and artificial disasters and resist decomposition for up to seven days following death^13–15^. Moreover, palatal rugae have been used as reference points for the position of the tongue during deglutition^16,17^ and it is also worth mentioning that maximum tongue pressure and maximum swallowing tongue pressure have been measured on these specific anatomical structures^18^.

The most common methods to assess the morphology—but not the complexity—of individual palatal rugae are partly subjective and identify the traits of rugae in a qualitative way, regarding their shape characteristics (such as actual shape, direction and unification), or use ordinal variables, regarding their length (e.g.: primary, secondary or tertiary rugae)^1,19–23^. The differences in the indices used for the categorisation of the rugae as per their qualitative characteristics, combined with investigators’ subjectivity, may result in confusion and potential lack of repeatability among evaluators. Moreover, such methods do not take into consideration rugae that happen to be shorter than a specific length, and definitely do not express a measure of complexity, but just a description of the morphological characteristics of individual rugae. Therefore, a more objective and quantitative method could be useful for the evaluation of the complexity of rugae.

Fractal dimension analysis (FDA) is a mathematical method suitable for quantifying geometrical configurations that are too irregular to be described in classical ways, such as Euclidean geometry^24^, but at least display adequate fractal regularity^24,25^. Fractals became popular thanks to the work of Mandelbrot^26^. Following their dissemination^27,28^, the concepts of fractal geometry have been useful in the analysis of diverse natural phenomena and geographical features, such as clouds and coastlines^26,28^. FDA has also been implemented in medicine and dentistry. Specifically in dentistry, it has been employed for the assessment of alveolar bone microstructure, especially via periapical radiographs^29^, and the evaluation of osteoporosis^27^. Other applications include the assessment of peri-implant bone trabecular microstructure changes^30^, healing after root canal treatment and surgery, assessment of dental materials, caries and dental tissues^31^. However, to our knowledge, fractal analysis has not been applied in the study of the complexity of rugae in a systematic and detailed way, yet.

Therefore, our objective is to present a new method, based on fractal dimension analysis, for the assessment of the complexity of palatal rugae, on pre-treatment models of orthodontic patients, and to evaluate its inter- and intra-rater reliability.

## Methods and materials

### Ethical approval

All methods were carried out in accordance with relevant guidelines and regulations. The Research Ethics Committee of the Dental School of the National and Kapodistrian University of Athens approved the project and waived the need for informed patient consent, since it would be performed on anonymized dental models (Protocol number: 495/ 01.03.2022).

### Sample

Twenty randomly selected pre-treatment maxillary dental plaster models (type IV plaster, white colour) were retrieved from the archives of the Postgraduate Orthodontic Clinic of the National and Kapodistrian University of Athens. The selected models belonged to male and female patients, treated between 2000 and 2021, in the late mixed or permanent dentition, with a full complement of teeth and with less than 4 mm of dental crowding. Dental casts with artifacts, or of patients with palatally impacted teeth, cleft lip and/or palate, or patients who had undergone a surgical procedure in the maxillary/ palatal region, were excluded.

### 3D model acquisition

The maxillary casts were scanned using a 3D structured white light scanner (Identica, Merit Co. Ltd, Seoul, South Korea) in order to obtain triangular mesh surface models.

### Digitisation and analysis of the 3D models

The digitised 3D maxillary models were imported and analysed with the Viewbox 4 software (dHAL Software, Kifissia, Greece). The following methodological steps were followed (software-specific details in Appendix in the supplementary material, Figs. 1 and 2):

1. Orientation: The digital model was oriented to align the transverse, sagittal and vertical directions with the software’s X-, Y- and Z- axes, respectively, where the X- and Y-axes are parallel to the occlusal plane (this convenience step is optional and does not affect the results).
2. Initial cropping: The area that contains rugae was encircled by connecting the cervical margins of the first molars, at the palatal grooves, to each other and with the most palatal points of all the teeth located mesial to them. The remainder of the mesh was deleted.
3. Creation of a smoothened surface (BP surface), without terrain anomalies, using the ball pivoting algorithm (BPA)^32,33^ (see below).
4. Distance mapping: Heights between the original mesh and the BP surface were computed at each vertex. Terrain features, detected as height differences between the original mesh and the BP surface, were depicted as a coloured texture on the original mesh.
5. Creation of contour lines: Based on the vertex distances from the BP surface, contour lines of the rugae were constructed on the mesh. The contour lines at a height of 0.15 mm were considered to represent the borderlines between the rugae and the remaining palatal surface. Each contour line was defined by points (Discrete points—DP) connecting edges of the triangular mesh surface.
6. Flattening of the surface: In the present method, Boundary First Flattening (BFF), a linear method for conformal parameterization, was used^34^.
7. Final cropping: Any contour lines that did not depict rugae, but adjacent anatomical structures, such as the incisive papilla and palatal gingivae, were removed.
8. Box-counting fractal dimension analysis (see below).

**Figure 1:**
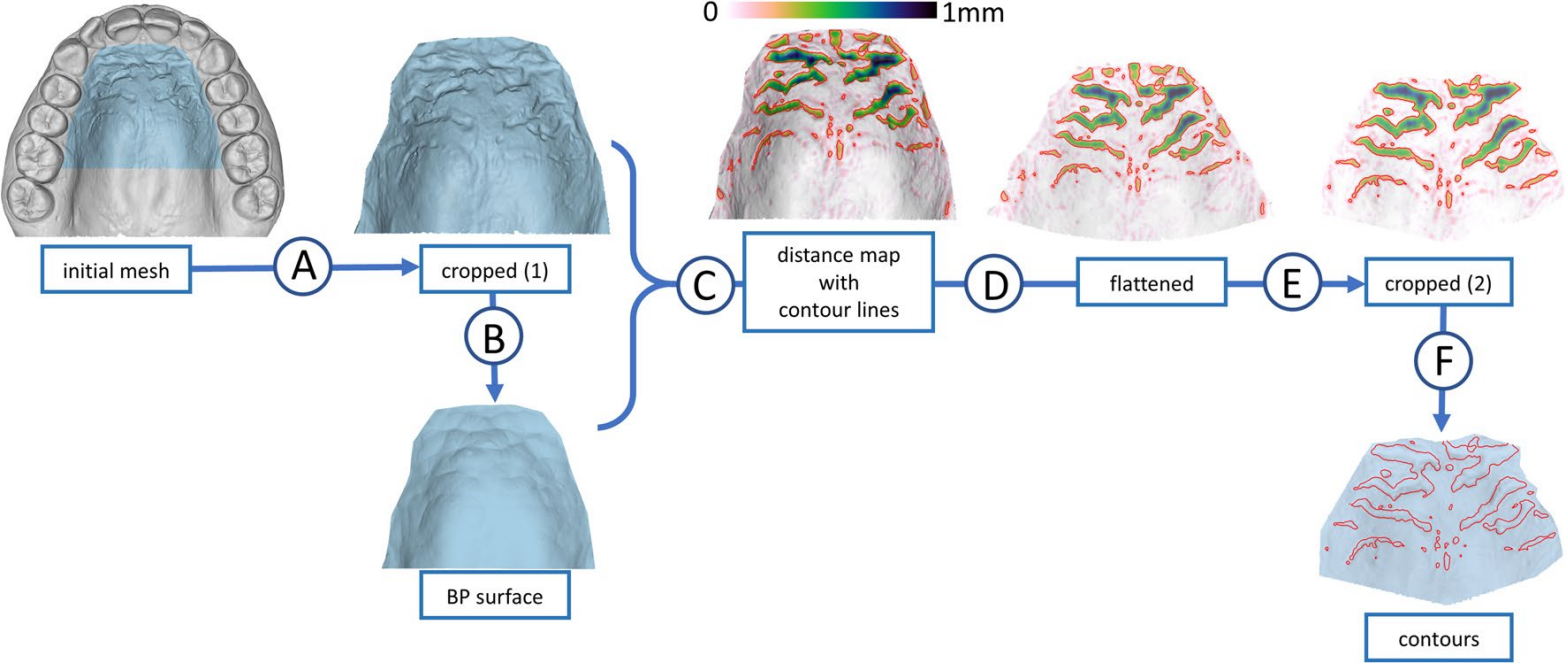
Steps of the method. (A) Initial cropping; (B) ball pivoting; (C) distance mapping and creation of isolines (contour lines); (D) flattening; (E) final cropping; (F) removal of the texture. BP surface, ball pivoting surface.

**Figure 2:**
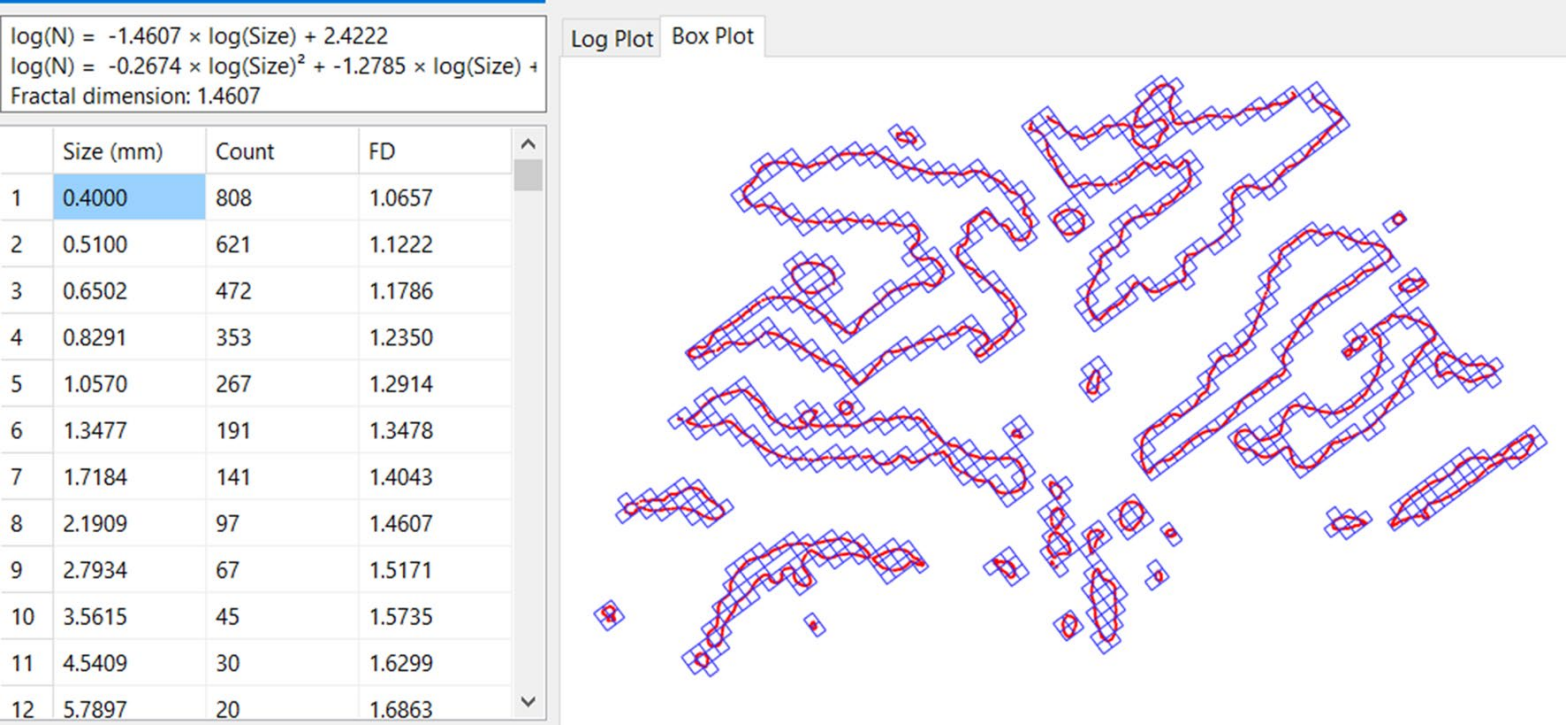
Box-counting fractal dimension analysis.

### Ball pivoting algorithm

The Ball Pivoting algorithm (BPA) is applied as a prerequisite for distance mapping. It creates a smoothened surface mesh [ball pivoting surface (BP surface)] based on the original mesh, by rolling a virtual “ball” over it^32,33^. In this investigation, a modified version of the mesh-aware ball-pivoting algorithm^33^, available in Viewbox 4, was employed.

The most important factor of the ball pivoting procedure, and the only parameter determined by the operator, is the size of the ball’s radius. The radius needs to be of adequate size to smoothen the terrain anomalies of interest (palatal rugae) but not too large as to bridge over the curvature of the palate. If a larger than the optimal radius is used, smoothening of areas that do not constitute rugae occurs. The ball is not able to follow the curvature of the palate, and the detected distance between the BP surface and the original mesh appears greater, resulting in overestimation of the width and height of the rugae. On the other hand, if a smaller ball radius is used, the ball pivots inside the rugae leading to underestimation of their size and height.

Following a series of trials with different radii ranging from 0.5 to 20 mm, we determined that the ball radius should range between 3 and 4 mm, in accordance with the rugae’s scale. A 3.5 mm ball radius was set for all experiments of the current study.

### Box-counting fractal dimension analysis

Box-counting fractal analysis is a sampling process used to calculate the box-counting fractal dimension^35^. A series of grids of decreasing calibre (the so-called “boxes”) is systematically placed over an image. Data recording (the so-called “counting”) is performed for each calibre and represents the minimum number of boxes in each grid that contain any part of the important detail (contour of palatal rugae in our case) in them^35^.

For reliable estimation of the fractal dimension, several parameters need to be set. The minimum and maximum box sizes define the limits within which box sizes will range. The number of box sizes refers to the number of grids, as each grid is made of boxes of one size only. Finally, the number of search positions corresponds to the relative positions between the grid and the image. The following values were used in this study: minimum box size: 0.4 mm; maximum box size: 12 mm; number of box sizes: 15; number of search positions: 15 for translation and 45 for rotation around the Z axis (see Appendix in the supplementary material).

### Statistical analysis

The measurement procedure was performed independently by two of the authors (MAM, DJH) and repeated after a 15-day interval by the first author (MAM). Bland–Altman analysis was used to evaluate intra- and inter-rater agreement. Calibration between the raters, regarding the initial and final mesh cropping procedures, took place beforehand.

## Results

### Inter- and intra-rater reliability

**Figure 3:**
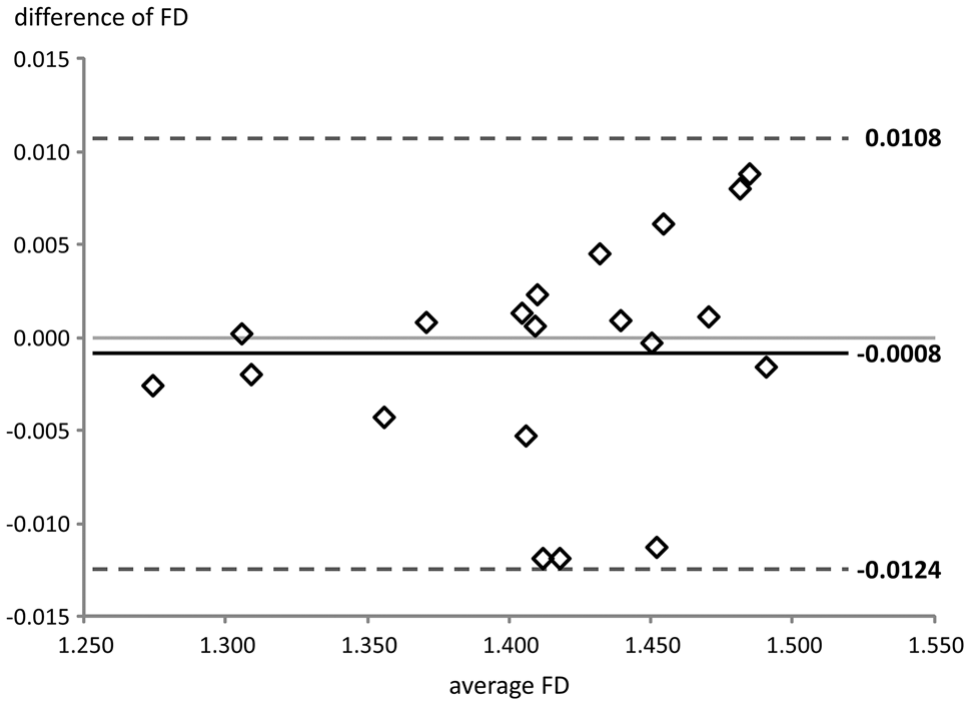
Bland–Altman plot for inter-rater agreement. FD, fractal dimension.

**Figure 4:**
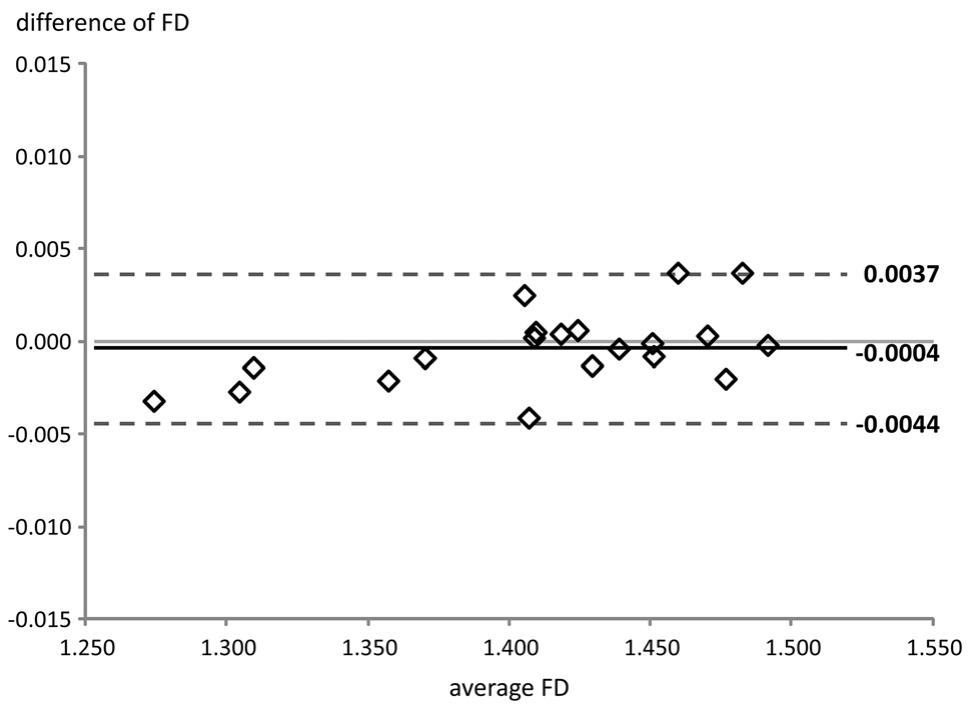
Bland–Altman plot for intra-rater agreement. FD, fractal dimension.

Fractal dimensions ranged from 1.274 to 1.491 (average: 1.412, standard deviation: 0.061). The Bland–Altman analysis of inter-and intra-examiner reliability demonstrated that the 95% limits of agreement ranged from − 0.012 to 0.011 and from − 0.004 to 0.004, respectively (Tables 1 & 2, Figs. 3 & 4). The 95% limits of agreement spanned from − 6 to 5% of the range of the values of the fractal dimensions for inter-rater reliability and from − 2 to 2% for intra-rater reliability.

**Table 1:** Fractal dimensions (FD), as measured by rater 1 (R1) and rater 2 (R2) (inter-rater agreement).

Model no	FD (R1)	FD (R2)	Average	Difference
1	1.4578	1.4465	1.4522	− 0.0113
2	1.4806	1.4894	1.4850	0.0088
3	1.3057	1.3059	1.3058	0.0002
4	1.4040	1.4053	1.4047	0.0013
5	1.3101	1.3081	1.3091	− 0.0020
6	1.4699	1.4710	1.4705	0.0011
7	1.4089	1.4112	1.4101	0.0023
8	1.4776	1.4856	1.4816	0.0080
9	1.4239	1.4120	1.4180	− 0.0119
10	1.4916	1.4900	1.4908	− 0.0016
11	1.4087	1.4034	1.4061	− 0.0053
12	1.2757	1.2731	1.2744	− 0.0026
13	1.4180	1.4061	1.4121	− 0.0119
14	1.4299	1.4344	1.4322	0.0045
15	1.4390	1.4399	1.4395	0.0009
16	1.4090	1.4096	1.4093	0.0006
17	1.4515	1.4576	1.4546	0.0061
18	1.3581	1.3538	1.3560	− 0.0043
19	1.3705	1.3713	1.3709	0.0008
20	1.4506	1.4503	1.4505	− 0.0003
Average	1.4121	1.4112	1.4116	− 0.0008
SD	0.0607	0.0622	0.0614	0.0059

**Table 2:** Fractal dimensions (FD), as measured by rater 1 (R1) at baseline (T0) and at T1 = T0 + 15 days (intra-rater agreement).

Model no	FD (R1) at T0	FD (R1) at T1	Average	Difference
1	1.4578	1.4615	1.4597	0.0037
2	1.4806	1.4843	1.4825	0.0037
3	1.3057	1.3030	1.3044	− 0.0027
4	1.4040	1.4065	1.4053	0.0025
5	1.3101	1.3087	1.3094	− 0.0014
6	1.4699	1.4702	1.4701	0.0003
7	1.4089	1.4048	1.4069	− 0.0041
8	1.4776	1.4756	1.4766	− 0.0020
9	1.4239	1.4245	1.4242	0.0006
10	1.4916	1.4914	1.4915	− 0.0002
11	1.4087	1.4089	1.4088	0.0002
12	1.2757	1.2725	1.2741	− 0.0032
13	1.4180	1.4184	1.4182	0.0004
14	1.4299	1.4286	1.4293	− 0.0013
15	1.4390	1.4386	1.4388	− 0.0004
16	1.4090	1.4095	1.4093	0.0005
17	1.4515	1.4507	1.4511	− 0.0008
18	1.3581	1.3560	1.3571	− 0.0021
19	1.3705	1.3696	1.3701	− 0.0009
20	1.4506	1.4505	1.4506	− 0.0001
Average	1.4121	1.4117	1.4119	− 0.0004
SD	0.0607	0.0618	0.0613	0.0021

The number of mesh triangles and vertices ranged approximately from 80,000 to 140,000 and from 160,000 to 270,000, respectively. Discrete points (DP) of the outlines of the rugae varied between 4,500 and 10,000.

## Discussion

Mandelbrot popularised fractal geometry in the 1980s^26^. Since then, various fractal analyses have been described in the literature. Amongst them, the box counting method has been implemented to determine the fractal dimension of shorelines, vegetation, river networks and fracture patterns^36^ and to calculate the area of irregular cartographic features^37,38^. Given its applicability with equal effectiveness to point sets, linear features, areas, and volumes, the box counting method is widely used for determining fractal dimensions^36^. We are aware of only one publication where fractal dimension has been used for palatal rugae^39^, however only for the purpose of image registration and not for the estimation of the complexity of rugae in a systematic and well-defined manner.

Studies that have assessed rugae have used methods which are descriptive of the size (described with ordinal variables), shape, branching or direction of individual rugae, and not the entire set. In contrast, the presented method is capable of measuring the complexity of either a single ruga, a selection of rugae or the entire set of a palate’s rugae, depending on which structures are preserved at the steps of Initial and Final cropping.

As far as box-counting fractal analysis is concerned, all recommendations by Kenkel^40^ were taken into account for the calculation of the rugae’s fractal dimensions. First, the outlines of the rugae were converted to point coordinates, instead of lines or pixels, and the number of points was well above the minimal recommended limit of 2,500^40^. In order to achieve a reliable estimation of the box count dimension, the minimum box size was set at 0.4 mm, ensuring that the box count was lower than M/10, where M: the number of points. Likewise, regarding the maximum box size, it was defined in such a way that all box sizes r, for which N(r) = (1/r)^2^, were excluded. Furthermore, 15 different box sizes were used, in accordance with Kenkel’s proposal that at least 10 should be employed, and the log–log plots were visually checked for departures from linearity^40^. Finally, in an effort to minimise the quantisation error^25^, more than 10,000 different search positions were employed (specifically 10,125 search positions; 15 translations per axis and 45 rotations).

The proposed method is a 2D-3D hybrid. It starts with cropping of the 3D palatal surface followed by the steps of construction of a BP surface, distance mapping and creation of rugae’s contour lines. The “Flattening” stage is the turning point from 3 to 2D. It could be omitted if the method was exclusively 3D and subsequently, the final step would be a 3D fractal dimension analysis, namely 3D box-counting (also known as cube-counting) fractal analysis^41^. However, if fractal dimensions were calculated with cube-counting, the shape of the palatal vault would influence the final outcome. As our objective was to evaluate the complexity of palatal rugae, independently of other parameters, flattening was considered necessary.

Flattening a 3D surface to a plane necessitates, in general, some distortion. Flattening algorithms can be conformal, minimising angular distortion, equiareal, minimising distortion of areas, or can minimise some combination of angle and area distortion^42^. Conformal flattening is advantageous for the study of rugae. Nevertheless, existing methods either offer little direct control over the shape of the flattened surface or demand significant nonlinear optimization^34^. In the present method, Boundary First Flattening (BFF) was used^34^. This is a linear method for conformal parameterisation which is faster than traditional linear methods. It “unfolds” and flattens the rugae area of the palate automatically and offers accurate preservation of sharp corners^34^.

Inter- and intra-rater repeatabilities were high due to minimal human interaction and operator calibration. Only two of the steps require subjective action by the operators. The step of “Initial cropping” determines the area that will be flattened and therefore affects the mesh distortion that will occur. The “Final cropping” step determines which of the structures should be considered as rugae. This stage is inevitable in all existing methods to distinguish which structures are to be measured or evaluated according to their shape, direction or branching. Contrary to previous qualitative methods, where the raters need to be calibrated in regard to the qualitative indices, in the present method, calibration is necessary only for the distinction of the contours that correspond to rugae.

As expected, the limits of agreement for intra-rater reliability were narrower than those for inter-rater agreement. Also, both inter- and intra-observer errors increased with fractal dimension (Figs. 3 and 4). A logical explanation is that as the complexity/fractal dimension increases, it becomes harder for the operator to differentiate rugae from other adjacent anatomical structures, such as incisive papilla and palatal gingivae, however, without having a great impact on the final result.

Advantages of the proposed method include a comprehensive evaluation of the complexity of all rugae (regardless of their size), with fractal analysis and a complete set of information about their contours and heights, with distance mapping. Minimum user intervention is needed, rendering the results repeatable and objective. In addition, fractal dimensions are quantitative variables and this makes them comparable.

Limitations of the method include sensitivity to parameters, such as ball radius and selection area. The size of the ball radius could over- or underestimate the size of rugae, and therefore their fractal dimensions, as well. Moreover, distortion is unavoidable after mesh flattening. The selected area could affect the extent of distortion, and rugae’s fractal dimension. Therefore, the operators need to be calibrated as per area selection.

Another drawback is the use of fractal dimension as a single measure of complexity. Despite its advantages, this method does not provide any information about the qualitative characteristics of rugae, such as shape, position, direction or branching or their quantitative variables, such as width, length or number.

Applications of this method could be helpful in forensics and dental research. With regard to the former, the procedure of ante- or post-mortem identification can be automated by means of distance mapping. Distance mapping is an intermediate stage of our method, as fractal dimensions are calculated in the end. However, in the case of identification, this step may be sufficient. Our method, which is a 2D-3D hybrid, could outweigh other exclusively 2D imaging methods^19–23^, as it provides information about the height of all rugae and details difficult to be detected otherwise. Undoubtedly, fractal analysis could be also used for the purpose of identification, by comparing rugae’s fractal dimensions in different sets of records. Both distance mapping and fractal analysis can be also applied partially, if the entire palate is not available.

As far as dental research is concerned, this method could be applied in various topics of research. First of all, it could be used to investigate a potential association of the complexity of palatal rugae and other anatomical structures, such as the size and number of teeth or the shape of the palate^43^, which may have common genetic background with rugae^44^. It could also detect potential correlations between specific genes and the complexity of the rugae, or help identify genetic polymorphisms or developmental stress—that could lead to asymmetries^45^—in the formation of rugae and provide insights into the hypothesis that palatal rugae are formed by a reaction–diffusion mechanism^4^. Furthermore, it has been proposed that rugae may change following various interventions (maxillary expansion, tooth extractions, etc.)^46–50^. Fractal dimension could be employed to exhibit the robustness of this hypothesis. Besides, since rugae participate in mastication and deglutition^7^, this method could be used to disclose potential relationships. Moreover, distance-mapped rugae could be used for improving superimpositions of stable regions of palate for measuring tooth movements (e.g. before and after orthodontic treatment).

Finally, this study was performed on digitised scanned plaster models, as the objective was to develop the methodology and to examine its reliability. Should it be implemented for one of the aforementioned purposes, it is recommended that it be used on direct scans of the rugae in order to minimise potential errors and problems related to dimensional changes of the impression materials and gypsum^51,52^.

## Conclusions

The novel method presented in this report, constitutes a sequence of steps that are performed with various features available in the software used. The method has been shown to be reliable according to the inter- and intra-rater agreements and can be used as a quantitative, objective method of comprehensive assessment of the complexity of palatal rugae. It necessitates minimum user intervention and it can be applicable in forensics and dental research.

## Supplementary Information

Supplementary Information.

## Data Availability

The datasets generated and/or analysed during the current study are available on request from the corresponding author.
