# Supplementary material for: Novel method to delineate palatal rugae and assess their complexity using fractal analysis
Source: Sci Rep. 2022 Dec 16;12:21749. doi: 10.1038/s41598-022-25910-y (PMC9758149; doi:10.1038/s41598-022-25910-y)
Supplement: Supplementary file 1 — Supplementary Information. [file 41598_2022_25910_MOESM1_ESM.pdf]

Appendix to "Novel method to delineate palatal rugae and assess their complexity using fractal analysis". Technical details about the method (Figures 1 and 2)

All steps were performed in Viewbox 4 (dHAL software, Kifissia, Greece), pre-release version 4.1.0.14:

1. Orientation: The digital model is oriented using the "ground plane" feature in Viewbox 4, which automatically aligns the occlusal plane to the X-Y plane of the coordinate system. Manual rotation around the Z axis brings the palatal midline in alignment with the Y axis (this convenience step is optional and does not affect the results).
2. Initial cropping: The area that contains the rugae is selected using the "Area Select" tool. Selection starts from the point where the palatal groove of the right first molar meets its cervical margin, continues to the corresponding point of the left molar and then to the most palatal points of the teeth located mesial to the first molars. In this way, the whole rugae-containing area is selected. The rest of the mesh is deleted using "Delete inverted selection".
3. Ball pivoting: A version of the ball pivoting (BP) algorithm is applied with a radius of 3.5 mm on the back side of the selected mesh, using the Ball Pivoting command from the Mesh functions menu. A new surface (BP surface) is created and set as the reference mesh.
4. Distance mapping: The distance of each vertex to the BP surface is computed using the "Distance map" function and assigned to the corresponding vertex via the "Set distance as vertex attribute" command. Distances can be

converted to a colour map via a lookup table (LUT) and assigned to the mesh as a texture.

5. Creation of contour lines: Contour lines are created by selecting "Create Isolines" from the "Vertex Attributes" menu. A threshold of -0.15 mm is set, representing the outlines of the rugae (the value is negative because the BP surface was constructed on the back side of the original surface). Contour lines are composed of many small line segments that join points located on the mesh at the height set by the threshold. The number of points, and line segments, is related to the resolution of the mesh surface.
6. Flattening of the surface: The mesh is flattened using the "Flatten" command from the "Modify vertices" menu. We used the Iterative Cholesky solver ("ICholesky") from the available options.
7. Final cropping: Any non-rugae contour lines are deleted using the "Select area" tool, followed by "Delete selection".
8. Box counting fractal analysis: From the "Tools" menu, "Box count" is selected. It gives the option to customize the minimum and maximum box sizes, as well as the number of box sizes and search positions. To follow the recommendations suggested by Kenkel (2013), these values were used:

   Minimum box size: 0.4 mm

   Maximum box size: 12 mm

   Number of box sizes: 15

   Number of search positions (translation): 15 (XY direction selected)

   Number of search positions (rotation): 45 (Z axis only selected)
